# A case of small bowel aneurysm hemorrhage with submucosal tumor‐like findings

**DOI:** 10.1002/deo2.335

**Published:** 2024-01-22

**Authors:** Susumu Banjoya, Yohei Minato, Yoshiaki Kimoto, Yuki Kano, Takashi Sakuno, Kohei Ono, Marie Osawa, Hajime Horiuchi, Teppei Morikawa, Ken Ohata

**Affiliations:** ^1^ Department of Gastrointestinal Endoscopy NTT Medical Center Tokyo Tokyo Japan; ^2^ Department of Radiology NTT Medical Center Tokyo Tokyo Japan; ^3^ Department of Diagnostic Pathology NTT Medical Center Tokyo Tokyo Japan

**Keywords:** double‐balloon endoscopy, small bowel resection, small intestine, submucosal aneurysm, submucosal tumor

## Abstract

A 51‐year‐old woman visited our hospital with the chief complaint of tarry stools. Contrast‐enhanced abdominal computed tomography revealed leakage of contrast medium into the lumen of the small intestine. Subsequently, a double‐balloon endoscopy was performed, which revealed a submucosal mass‐like lesion in the jejunum. Although hemostasis was attempted with clips, complete hemostasis was difficult to achieve, and angiographic embolization was performed. Nevertheless, the anemia progressed, and a small bowel resection was performed. Histopathological examination led to a diagnosis of a ruptured submucosal aneurysm of the small intestine. Endoscopic hemostasis is often difficult to achieve for submucosal aneurysms in the intestine. The submucosal tumor‐like finding observed on endoscopy in submucosal aneurysms is termed an “SMT‐like sign” and is considered an important finding to diagnose aneurysms.

## INTRODUCTION

Small intestinal bleeding is considered a relatively rare cause of gastrointestinal hemorrhage.^1^ Recent advances in endoscopic techniques have revealed small intestinal bleeding as a relatively frequent cause in cases of gastrointestinal bleeding of unknown cause.^2^ Despite this background, reports of ruptured submucosal aneurysms of the small intestine remain few, with only one reported case, to the best of our knowledge.^3^ We present a rare case of ruptured submucosal aneurysm in the small intestine, which was detected by double‐balloon endoscopy.

## CASE REPORT

**FIGURE 1:**
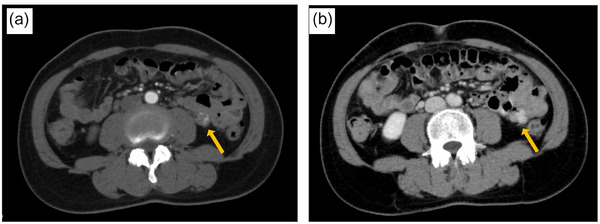
Contrast‐enhanced computed tomography (CT). (a) Arterial phase of contrast‐enhanced computed tomography showed contrast leakage into the lumen of the small intestine. (b) The late phase showed an increase in leaked contrast.

**FIGURE 2:**
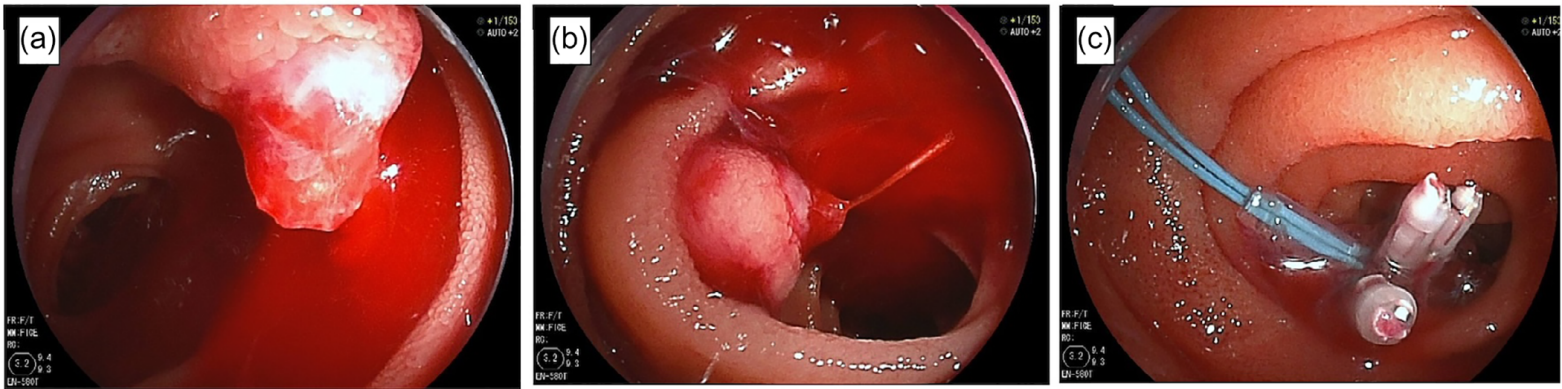
Findings of double‐balloon enteroscopy (DBE). A submucosal tumor‐like lesion with pulsatile bleeding in the jejunum. Hemostasis was attempted with a clip and an indwelling snare, but we were unable to stop the bleeding.

**FIGURE 3:**
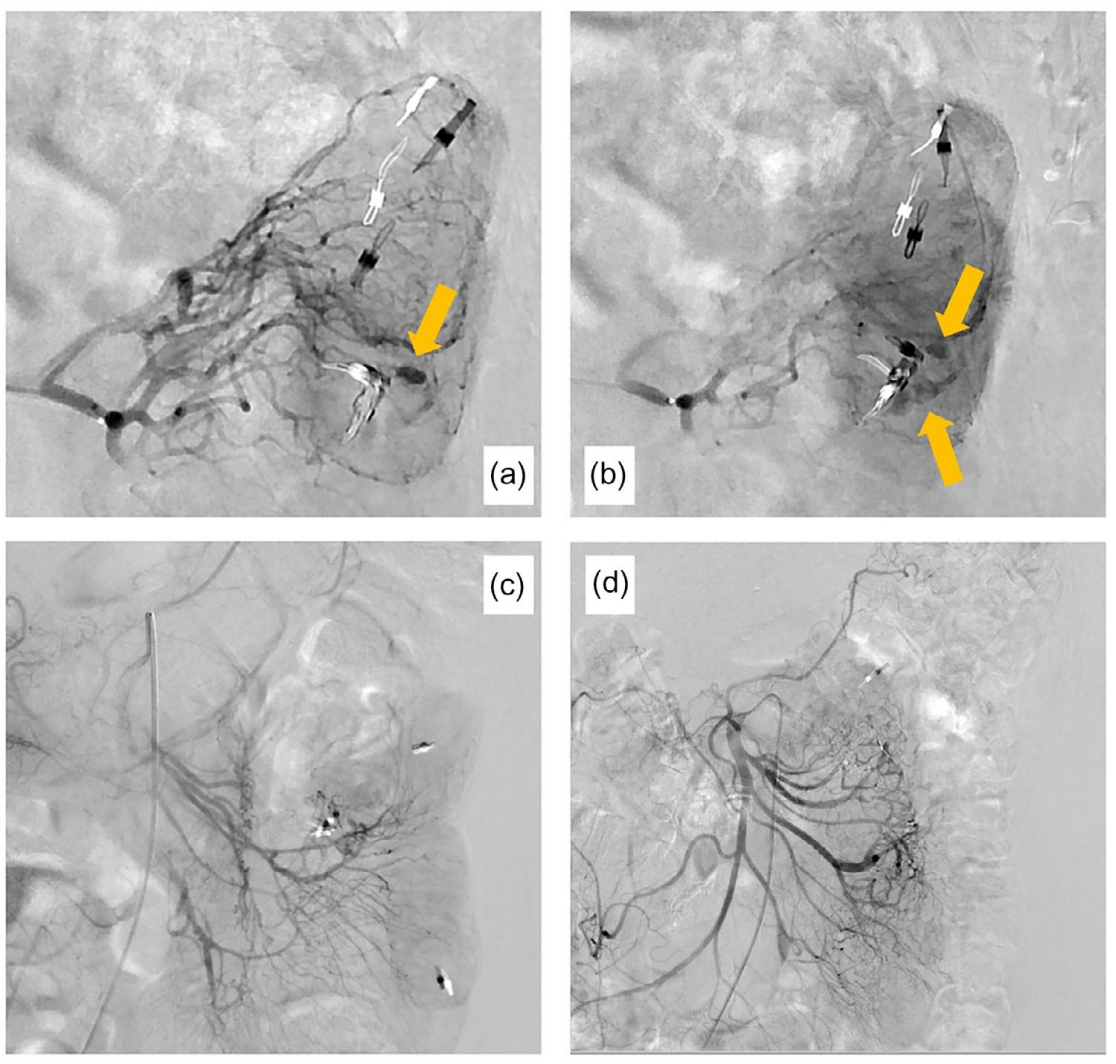
Angiography. (a, b) Confirming leakage of contrast from the distal end of the second jejunal artery into the intestinal lumen. The artery was embolized using *n*‐butyl‐2‐cyanoacrylate and Lipiodol. (c, d) The disappearance of extravascular leakage of contrast was confirmed.

**FIGURE 4:**
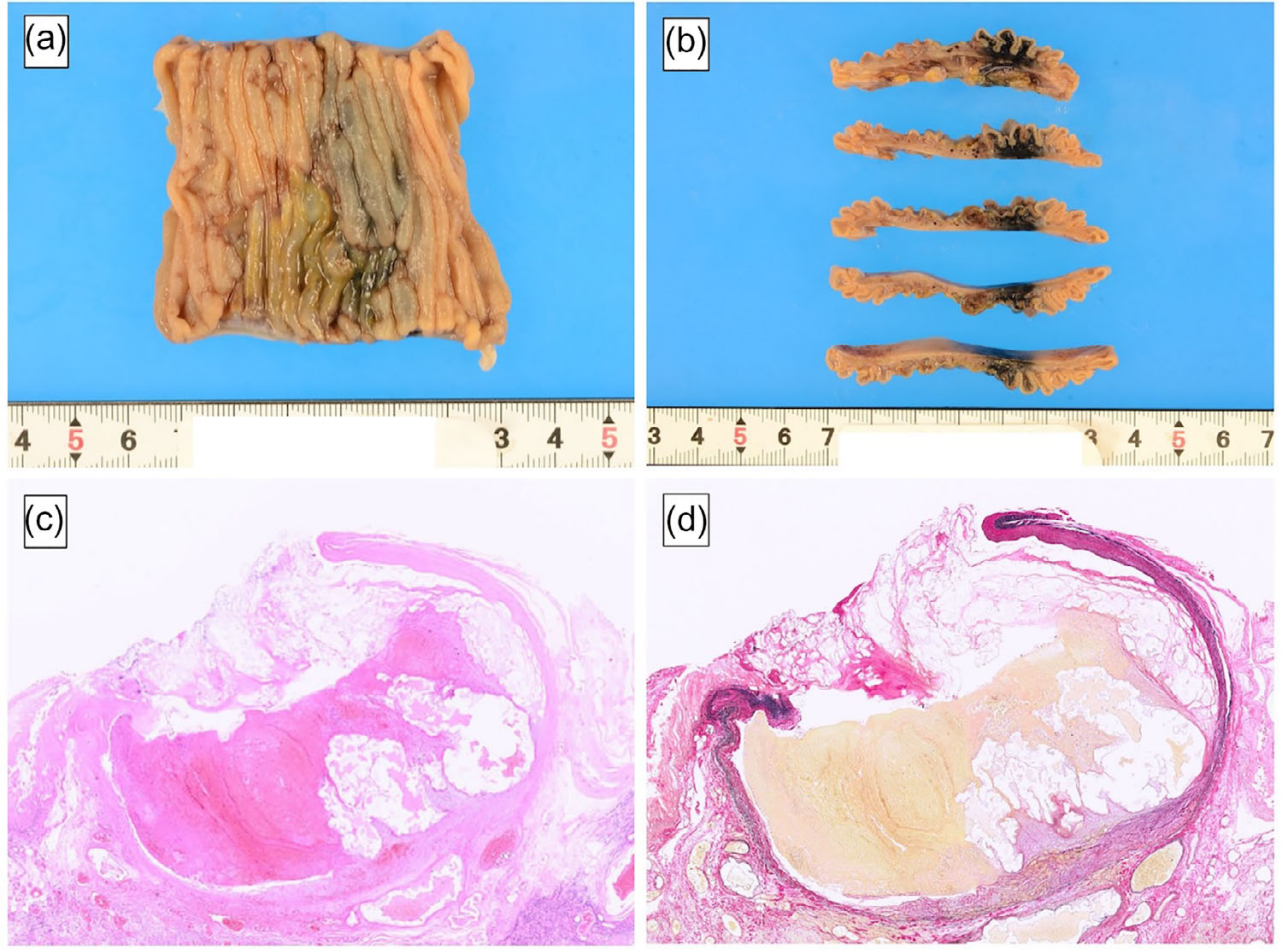
Surgical specimen. (a, b) surgical specimen. A huge dilated artery was present within the submucosa. The area around the aneurysm was necrotic, which was thought to be the result of arterial embolization. (c) Hematoxylin and eosin staining. (d) Elastica van Gieson staining. Histopathological examination revealed a submucosal large dilated artery. There was necrosis around the artery. The lumen of the vessel was filled with acidophilic non‐structural material. The mucosal to submucosal layers were widely obliterated, exposing the vessels directly into the mucosa and showing disruption of the vessel wall. Elastic fibers in the tunica media were highly reduced and almost disappeared. Pathological findings revealed a diagnosis of a ruptured submucosal aneurysm of the small intestine.

A 51‐year‐old woman visited our hospital with the chief complaint of lightheadedness, weakness, and tarry stool; she had been noticing bloody stools for about 5 days. She gave a history of having undergone ovarian cyst surgery. There was no history of allergies or receiving any oral medications. On admission, her vital signs were stable, with a blood pressure of 122/66 mmHg, a pulse rate was 84 beats/min, and a body temperature of 37.1°C. The laboratory findings were as follows: hemoglobin (Hb) 7.5 g/dl, red blood cell 7800/μL, hematocrit 21.9%, blood urea nitrogen 28.2mg/dL, and creatinine 0.62mg/dL. Physical examination revealed palpebral conjunctival pallor and no abdominal tenderness. The origin of the bleeding could not be identified by Esophagogastroduodenoscopy. Contrast‐enhanced computed tomography showed contrast leakage into the lumen of the small intestine, indicative of active hemorrhage. No masses or other abnormalities were observed (Figure 1a,b). On day 1 of admission, the Hb was 7.4 g/dL and 4 units of red blood cells (RBCs) were transfused. On day 2 of admission, the Hb was 7.1 g/dL and 6 units of RBCs were transfused. Then, given the suspicion of small intestinal bleeding, an antegrade double‐balloon endoscopy was performed, which revealed a submucosal tumor‐like lesion with pulsatile bleeding in the jejunum. Because there was no surrounding venous dilatation with active bleeding, we determined that Type 2 of the Yano Yamamoto classification was the most similar and indicated endoscopic hemostasis. Hemostasis with clips and a detachable snare proved unsuccessful (Figure 2). The examination was terminated after tattooing near the hemorrhagic lesion. While emergency surgery was considered, angiography was first performed for hemostasis. After confirming the leakage of contrast from the distal end of the second jejunal artery into the intestinal lumen, the artery was embolized using *n*‐butyl‐2‐cyanoacrylate (NBCA) and Lipiodol, and the disappearance of extravasation of the contrast was confirmed (Figure 3). As hemostasis was successfully achieved by arterial embolization, we could avoid surgical intervention. On day 3 of hospitalization, the patient's morning Hb level was 8.2 g/dL, but dropped to 7.5 g/dL in the evening, raising the suspicion of recurrent bleeding. The patient complained of abdominal pain with suspected intestinal ischemia due to arterial embolization, and there was concern that the bleeding from the aneurysm had not stopped due to continued bloody stools and worsening anemia. So, we performed surgery and laparoscopic small bowel resection on day 4 of admission. The tattoo was identified 30 cm from the ligament of Treitz on the anorectal side. The ischemic changes observed in the tattooed area were thought to be due to arterial embolization. A 5‐cm section of the small intestine, including the ischemic site, was resected (Figure 4a,b). Histopathological examination revealed a submucosal large, dilated artery with necrosis around it, likely a result of arterial embolization. The mucosal to submucosal layers were widely obliterated, exposing the vessel directly to the mucosa, with disruption of the vessel wall. Elastica van Gieson staining showed that the density of elastic fibers in the tunica media was markedly reduced and the fibers had almost disappeared in some areas. There were no findings suggestive of anastomosis between artery and vein, which is observed in arteriovenous malformations and other arterial abnormalities. Pathological findings revealed a diagnosis of a ruptured submucosal aneurysm of the small intestine (Figure 4c,d). The patient's postoperative recovery was uneventful, and she was discharged on day 10 of hospitalization. At present, more than 2 years after discharge, she remains under observation, with no evidence of recurrence to date.

## DISCUSSION

In the classification of endoscopic findings and treatment guidelines for small‐intestinal vascular lesions by Yano et al, the lesions are roughly classified into four categories based on the gross findings.^4^ Type 1 is an erythematous lesion of a few millimeters in size and is considered to be caused by vasodilatation. Type 2 is an arterial lesion, classified as type 2a and type 2b based on the presence or absence of projections; Dieulafoy lesions fall in this category. Type 3 lesions are pulsatile bulging lesions with surrounding venous dilatation and are considered arteriovenous malformations. Type 4 lesions are those lesions that cannot be classified under Types 1–3. The gross findings of submucosal aneurysms of the small intestine, including in our case and the previously reported case, correspond to a Type 2a or 2b lesion in this classification and can be confused with a Dieulafoy lesion. The recommended treatment is mechanical hemostasis with clip placement. However, in practice, hemostasis with clips is difficult, and in both the reported cases, including ours, partial resection of the small intestine eventually became necessary. Ruptured submucosal aneurysms should be positioned as Type 4 lesions, distinct from Dieulafoy lesions. Unlike Dieulafoy lesions, submucosal aneurysms are characterized grossly by the presence of a gently hemispherical mucosal ridge; we call this feature the “SMT‐like sign.” The larger size of submucosal tumor‐like elevation and the presence of pulsatile bleeding compared to the Dieulafoy lesion are considered characteristics of submucosal aneurysms. According to Mikó et al., Dieulafoy lesions had an average arterial diameter of 1080 μm,^5^ whereas the arterial diameter of the submucosal aneurysm of the small intestine we experienced was 10,000 μm, and we consider this may have influenced the difference in appearance of the size. Tannoury J et al. reported that a splenic aneurysm revealed a gastric submucosal tumor‐like lesion.^6^ We consider that the submucosal aneurysm we experienced in the small intestine appeared submucosal tumor‐like for the same reason as in previous reports. As far as we could find, there is only one case of a submucosal tumor‐like finding in a small intestinal submucosal aneurysm.^3^ We named it an “SMT‐like sign” because it is an important feature. Histopathological examination in our case showed saccular dilatation of the artery in the area where the “SMT‐like sign” was present. The patient had undergone endovascular treatment prior to surgery, and the possibility of accidental formation of a pseudoaneurysm was considered. Pseudoaneurysms are caused by trauma‐induced laceration of the vessel wall and subsequent formation of a peri‐arterial hematoma.^7^ In the resected specimen in our case presented herein, the layered structure of the artery was completely preserved, leading to the conclusion that it was a true aneurysm. It was assumed that the saccular dilatation of the artery extended under the normal mucosa, lifting up the normal mucosa, which was observed grossly as a submucosal mass.

Mikó et al. reported that the average arterial diameter of a Dieulafoy lesion was 1080 μm.^5^ On the other hand, the maximum diameter of the small‐intestinal submucosal aneurysm was 10,000 μm in our case and 3500 μm in the case previously reported by Chiba et al.^3^ Thus, small‐intestinal submucosal aneurysms are much larger in diameter, on average, than Dieulafoy lesions, which is presumably the reason why endoscopic hemostasis is difficult in cases of small‐intestinal submucosal aneurysms with bleeding. The clinical practice guidelines of the Society for Vascular Surgery recommend endovascular embolization for jejunal and ileal aneurysms, and when laparotomy is considered for hematoma removal and evaluation of bowel viability, open surgical ligation or resection of the aneurysms is recommended.^8^ In our case, endovascular embolization was performed first, but surgical treatment eventually became necessary when rebleeding was suspected. After partial resection of the small intestine, the bleeding did not recur again, so intestinal resection may be considered a promising treatment option for ruptured submucosal aneurysms of the small intestine with uncontrolled bleeding.

Ruptured submucosal aneurysms of the small intestine are often confused with hemorrhage from Dieulafoy lesions, but we would like to emphasize that the two are completely different clinical entities. Submucosal aneurysms of the small intestine are visualized as a submucosal tumor‐like mucosal elevation on endoscopy, which we consider useful for the diagnosis.

## CONFLICT OF INTEREST STATEMENT

None.

## ETHICS STATEMENT

All procedures followed have been performed in accordance with the ethical standards laid down Declaration of Helsinki and its later amendments.
